# Neutralization of *Clostridium difficile* toxin B with V_H_H-Fc fusions targeting the delivery and CROPs domains

**DOI:** 10.1371/journal.pone.0208978

**Published:** 2018-12-12

**Authors:** Greg Hussack, Shannon Ryan, Henk van Faassen, Martin Rossotti, C. Roger MacKenzie, Jamshid Tanha

**Affiliations:** 1 Human Health Therapeutics Research Centre, National Research Council Canada, Ottawa, Ontario, Canada; 2 Department of Biochemistry, Microbiology and Immunology, University of Ottawa, Ottawa, Ontario, Canada; 3 School of Environmental Sciences, University of Guelph, Guelph, Ontario, Canada; Cornell University, UNITED STATES

## Abstract

An increasing number of antibody-based therapies are being considered for controlling bacterial infections, including *Clostridium difficile* by targeting toxins A and B. In an effort to develop novel *C*. *difficile* immunotherapeutics, we previously isolated several single-domain antibodies (V_H_Hs) capable of toxin A neutralization through recognition of the extreme C-terminal combined repetitive oligopeptides (CROPs) domain, but failed at identifying neutralizing V_H_Hs that bound a similar region on toxin B. Here we report the isolation of a panel of 29 V_H_Hs targeting at least seven unique epitopes on a toxin B immunogen composed of a portion of the central delivery domain and the entire CROPs domain. Despite monovalent affinities as high as *K*_D_ = 70 pM, none of the V_H_Hs tested were capable of toxin B neutralization; however, modest toxin B inhibition was observed with V_H_H-V_H_H dimers and to a much greater extent with V_H_H-Fc fusions, reaching the neutralizing potency of the recently approved anti-toxin B monoclonal antibody bezlotoxumab in *in vitro* assays. Epitope binning revealed that several V_H_H-Fcs bound toxin B at sites distinct from the region recognized by bezlotoxumab, while other V_H_H-Fcs partially competed with bezlotoxumab for toxin binding. Therefore, the V_H_Hs described here are effective at toxin B neutralization when formatted as bivalent V_H_H-Fc fusions by targeting toxin B at regions both similar and distinct from the bezlotoxumab binding site.

## Introduction

*Clostridium difficile* is a Gram-positive spore-forming bacterium that continues to be a problematic nosocomial pathogen. The symptoms of gastrointestinal *C*. *difficile* infections can range from mild diarrhea to pseudomembrane colitis and death. The spore-forming nature of the pathogen coupled with an ability to rapidly colonize patients on broad-spectrum antibiotics presents a significant challenge to infection control in healthcare settings. Healthcare associated costs of managing *C*. *difficile* infection were estimated to exceed a staggering $4.5 billion annually in the US alone [1]. Despite the introduction of therapies that include new antibiotic modalities, fecal transplantation and antibody-based immunotherapy, efficacy limitations remain which necessitate the continuous search for more potent therapeutic agents [2,3,4].

*C*. *difficile* secretes two large toxins (TcdA and TcdB) that act upon the epithelial cells lining the gastrointestinal tract by first internalizing and then inactivating Rho/Ras proteins, which leads to cell-cytoskeleton disruption and ultimately a loss of epithelial barrier function, severe inflammation and apoptosis [5,6]. TcdA and TcdB are each four-domain proteins composed of an N-terminal glucosyltransferase domain, a central autoprotease cutting domain, a neighboring central delivery/translocation domain and a C-terminal region referred to as the combined repetitive oligopeptides (CROPs) domain. These two toxins, along with transferase toxin (CDT), are considered the primary virulence factors of *C*. *difficile* and have been targeted by toxin-binding polymers, vaccines and antibodies as strategies to control *C*. *difficile*-associated disease [5]. With respect to antibodies a number of anti-toxin formats have been explored, including intravenous immunoglobulin therapy, IgG, IgA, IgY, single-chain fragment variable (scFv) and single-domain antibodies (sdAbs) [2,7]. Recently, the anti-TcdB monoclonal antibody (mAb) bezlotoxumab was FDA-approved for the treatment of recurrent *C*. *difficile* infection [8,9].

In an effort to develop novel antibody-based therapeutics for *C*. *difficile* infection, our group and others have explored the use of sdAbs as potent anti-toxin neutralizing agents [10,11,12,13,14,15,16,17,18,19]. Camelid-sourced sdAbs (V_H_Hs or Nanobodies) are recombinant antibody fragments that offer the benefits of full-sized mAbs–high target affinity and specificity–with unique properties, most notably their amenability to tandem formatting in various geometries such that multi-specificities can be attained within a single molecule [20,21,22]. We previously isolated several moderate-affinity llama V_H_Hs that targeted the C-terminal CROPs domain (also referred to as the receptor binding domain or RBD) of TcdA (aa 2304–2710) that were neutralizers of TcdA as single V_H_Hs or in combination [10]. At the same time, immunization with a small C-terminal fragment of the TcdB CROPs domain (aa 2286–2366) failed to produce neutralizing V_H_Hs.

Here, we immunized a llama with a recombinant TcdB fragment (aa 1751–2366) that encompasses a portion of the central delivery/translocation domain (aa 1751–1833) and the entire CROPs domain (aa 1834–2366). We hypothesized that extending our previous immunogen design to include a portion of the delivery/translocation domain and the full-length CROPs domain may lead to neutralizing antibodies. This is supported by recent reports showing TcdB binding the frizzled (FZD2) family of Wnt receptors [23,24] and the poliovirus receptor-like 3 (PVRL3) receptor [25] through the central delivery/translocation domain, and TcdB binding the chondroitin sulfate proteoglycan 4 (CSPG4) receptor [26] through a region adjacent to the CROPs domain. In addition, several earlier studies showed the isolation of neutralizing antibodies to the CROPs region of TcdB [15,27,28]. Despite isolating numerous V_H_Hs with affinities as high as *K*_D_ = 70 pM, none of the monomeric antibodies were capable of preventing TcdB-induced cytotoxicity in cell-based assays. However, when reformatted as Fc-fusions, several V_H_H-Fcs were transformed into potent TcdB neutralizers on par with the neutralizing efficacy of the recently approved anti-TcdB mAb bezlotoxumab. Thus, TcdB-binding V_H_Hs targeting a fragment of the delivery domain and the complete CROPs domain are effective neutralizers when presented in the context of larger bivalent V_H_H-Fc fusions, presumably due to steric and/or avidity effects not afforded to monomeric V_H_Hs.

## Materials and methods

### Llama immunization, serum fractionation and serology

Recombinant TcdB (aa 1751–2366; hereafter referred to as TcdB_1751-2366_) was a generous gift from Dr. Kenneth Ng (University of Calgary, Calgary, AB, Canada). A llama was immunized with 100 μg of TcdB_1751-2366_ per injection, following a similar immunization schedule and adjuvanted as previously reported [29]. Serum from blood drawn 42 days post immunization was tested for binding to TcdB_1751-2366_ by ELISA essentially as described [10]. Serum was fractionated according to established protocols with protein G and protein A affinity columns [30] and conventional and heavy-chain IgG fractions tested for TcdB_1751-2366_ binding by ELISA [10]. The ability of polyclonal fractions to neutralize TcdB VPI 10463 (List Biological Laboratories) was examined by Vero cell toxin inhibition assays, essentially as described for TcdA [19] with minor modifications. Vero cell monolayers were incubated with a final TcdB concentration of 10 pM (2.7 ng/mL) or 30 pM (8.1 pg/mL) and 1 μM of day 42 fractionated serum for 72 h at 37°C and 5% CO_2_ before addition of WST-1 cytotoxicity reagent (Roche, Mississauga, ON, Canada) for 30 min and subsequent absorbance measurement at 450 nm.

### Research involving animals

All procedures involving llamas and their care were approved by the National Research Council Canada Animal Care Committee and by the Animal Care Committee of Cedarlane Laboratories who is licensed by the Ontario Ministry of Agriculture, Food and Rural Affairs.

### Library construction, V_H_H isolation and expression

Lymphocytes obtained from serum drawn 42 days post immunization served as a starting point for phagemid library construction. RNA extraction, cDNA synthesis, two rounds of PCR, restriction digestion, ligation into the pMED1 phagemid vector, transformation of electrocompetent *E*. *coli* and preparation of library phage were all performed as described [10,30]. V_H_Hs were then selected by two approaches. In the first approach, TcdB_1751-2366_ was coated directly onto microtiter plate wells, plates were blocked with 5% non-fat skimmed milk in PBS-T (PBS + 0.5% (v/v) Tween 20), and library phage applied, washed and eluted with 0.1 M triethylamine, all essentially as reported [10], for three rounds. In the second approach, TcdB_1751-2366_ was first biotinylated (TcdB_1751-2366_-Biotin) using a commercial EZ-Link^TM^ Sulfo-NHS-Biotinylation Kit (ThermoFisher, Ottawa, ON, Canada), according to the manufacturer’s instructions, and confirmed by Western blotting by probing with streptavidin (SA) conjugated with AP (ThermoFisher). Next, library phage were incubated with TcdB_1751-2366_-Biotin (5 nM) in a 1.5 mL Eppendorf tube for 10 min before addition of non-biotinylated TcdB_1751-2366_ competitor (2.5 μM) for 10 min. The mixture was then applied to streptavidin coated microtiter plates (ThermoFisher) for 5 min before a series of washes with PBS and PBS-T and elution with 0.1 M triethylamine. In each subsequent round the concentration of TcdB_1751-2366_-Biotin target was reduced and the incubation time with TcdB_1751-2366_ competitor increased, except for the fourth round in which the incubation time was held at 60 min. Eluted phage clones displaying V_H_Hs from both isolation methods were tested for binding to immobilized TcdB_1751-2366_ in monoclonal phage ELISA as described [10] and those clones producing the highest absorbance (450 nm) were sequenced, subcloned into the pSJF2H expression vector [10], and expressed and purified from *E*. *coli* by IMAC [10,30]. Purified V_H_Hs were probed by Western blotting using an α-His tag specific IgG conjugated with AP (ThemoFisher).

### V_H_H characterization

The aggregation state of V_H_Hs was assessed by size-exclusion chromatography (SEC) using a Superdex^TM^ 75 Increase column (GE Healthcare, Mississauga, ON, Canada) as described [10,31]. V_H_H thermal unfolding and refolding were examined by circular dichroism spectroscopy, essentially as reported [31], with the exception of data collection every 0.2°C and the V_H_Hs were allowed to cool at 25°C for 3 h before the second thermal melt was performed. The monovalent binding affinities of V_H_Hs were determined using a Biacore 3000 surface plasmon resonance (SPR) instrument (GE Healthcare) and the Biotin CAPture Kit (GE Healthcare). Approximately 900 resonance units (RUs) of TcdB_1751-2366_-Biotin were captured before flowing a two-fold dilution series of SEC-purified V_H_Hs (concentrations ranging from as low as 0.13–2 nM to as high as 31.3–500 nM) over the TcdB_1751-2366_-Biotin surface using single-cycle kinetic analysis. All experiments were performed in HBS-EP buffer (10 mM HEPES, pH 7.4, 150 mM NaCl, 3 mM EDTA, 0.005% (v/v) Surfactant P20; GE Healthcare) at 25°C at a flow rate of 30 μL/min, with a contact time of 2 min and dissociation time of 10 min. Surfaces were regenerated according to the Biotin CAPture Kit instructions (Regeneration stock 1 and 2, at 10 μL/min). Reference flow cell subtracted sensorgrams were fit to a 1:1 binding model using the BIAevaluation 4.1 software (GE Healthcare). V_H_H epitope binning was also performed by SPR on TcdB_1751-2366_-Biotin captured surfaces by injection of the first V_H_H at 10× *K*_D_ concentration for 2 min followed immediately by injection of a mixture of the first V_H_H + a second V_H_H at 10× *K*_D_ concentration for 2 min, all at a flow rate of 30 μL/min. Binning experiments were also performed in the reverse format (i.e., V_H_H2 followed by V_H_H2 + V_H_H1). The ability of V_H_Hs to neutralize TcdB VPI 10463 was examined by Vero cell toxin inhibition assays, essentially as described above for fractionated serum. Vero cell monolayers were incubated with a final TcdB concentration of 1 pM (270 pg/mL) and 1 μM of V_H_H for 72 h at 37°C and 5% CO_2_ before addition of WST-1 cytotoxicity reagent (Roche) for 30 min and subsequent absorbance measurement at 450 nm. Before performing TcdB inhibition assays that involved V_H_Hs, dimers and Fc-fusions, dose-response experiments were always conducted to identify a working TcdB concentration that provided ~90% toxicity to account for batch-to-batch variability in TcdB potency.

### Generation and characterization of V_H_H-V_H_H dimers

Using the three highest affinity V_H_Hs targeting unique epitopes (B39, B74 and B167) all V_H_H-V_H_H dimer formats were constructed by splice overlap extension PCR, essentially as described [31], before ligation into pSJF2H expression vector. Each dimer was separated by a 25 amino acid linker (Gly_4_Ser)_5_ and contained a C-terminal His_6_ tag for purification. Dimers were expressed in 1 L *E*. *coli* cultures, extracted from the periplasm by osmotic shock, purified by IMAC and assessed for purity and aggregation by SDS-PAGE, Western blot and SEC with a Superdex 200 Increase column (GE Healthcare), according to standard methods. When V_H_H-V_H_Hs showed higher order aggregates or signs of degradation by SEC, the non-aggregating monomer peaks were collected and used for subsequent assays. The thermal unfolding temperature of each dimer was determined as described above for monomer V_H_Hs. The dissociation rate of each V_H_H-V_H_H dimer was determined by SPR using conditions described above for binding to TcdB_1751-2366_-Biotin captured surfaces. Briefly, dimers and control monomers were injected at 1 nM for 5 min before 30 min of dissociation, all at 25°C with a flow rate of 30 μL/min and in HBS-EP buffer (GE Healthcare). Dissociation phases were fit to a 1:1 dissociation model using the BIAevaluation 4.1 software (GE Healthcare) in order to calculate *k*_d_s (s^-1^). The ability of V_H_H-V_H_H dimers to neutralize TcdB (List Biological Laboratories) was examined by Vero cell toxin inhibition assays as described above for fractionated serum and V_H_H monomers. A final TcdB concentration of 3 pM (810 pg/mL), based on dose-response experiments, and a dimer concentration of 1 μM were used. All other assay conditions remained the same. An irrelevant dimer T5/TC9 [32] was used at 1 μM as a negative control while the TcdB-binding mAb bezlotoxumab (MDX-1388) was used at 250 nM as a positive control.

### Generation and characterization of V_H_H-Fc fusions

V_H_Hs fused to the N-termini of human IgG1 Fcs were synthesized and subcloned into the pTT5 vector and expressed through transient transfection of mammalian HEK293-6E cells before protein A purification, as described [33,34]. Each V_H_H was separated from the IgG1 Fc region by either the human IgG1 hinge or a 35-residue camel/llama γ2a hinge [35]. The aggregation profiles of V_H_H-Fcs were assessed by SEC using a Superdex 200 column (GE Healthcare). SPR was used to determine the dissociation rates of V_H_H-Fcs by flowing 1 nM of SEC-purified antibody over TcdB_1751-2366_-Biotin surfaces for 2 min, followed by a dissociation time of 30 min, all at 25°C with a 30 μL/min flow rate in HBS-EP buffer (GE Healthcare). SPR-based binning experiments between V_H_H-Fcs and MDX-1388 [36] were performed essentially as described above for V_H_H monomers. The ability of V_H_H-Fcs to neutralize TcdB (List Biological Laboratories) was examined by Vero cell toxin inhibition assays as described above for V_H_H monomers and dimers. A final TcdB concentration of 500 fM (135 pg/mL), based on dose-response experiments, and a V_H_H-Fc fusion concentration of 250 nM were used. All other assay conditions remained the same. When two V_H_H-Fc fusions were used in combination for TcdB inhibition, 125 nM of each V_H_H-Fc was added. TcdB and V_H_H-Fcs were not pre-incubated before the addition to Vero cells.

## Results

### Llama immunization, serum fractionation and serology

**Fig 1:**
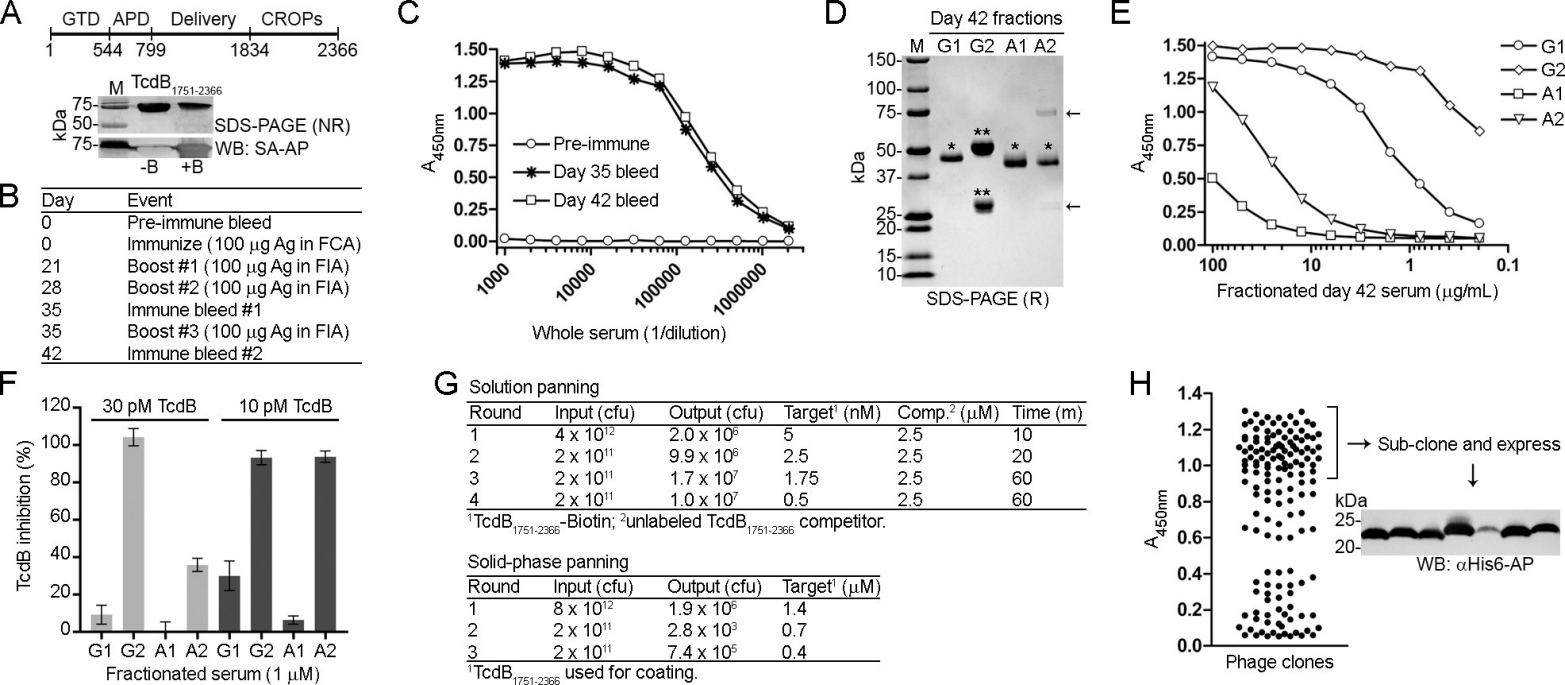
Isolation of high-affinity TcdB-binding V_H_Hs. (A, *top*) Schematic of TcdB. GTD, glucosyltransferase domain; APD, autoprotease domain, Delivery, delivery/receptor binding domain, CROPs, combined repetitive oligopeptides domain. (A, *bottom*) A 71.6 kDa fragment of *C*. *difficile* toxin B (TcdB_1751-2366_) was purified, biotinylated and analyzed by non-reducing (NR) SDS-PAGE and Western blot (WB) probed with streptavidin-AP (SA-AP). Approximately 1 μg of protein was loaded per lane. M, protein molecular weight marker; -B, unlabeled TcdB_1751-2366_; +B, biotinylated TcdB_1751-2366_. (B) TcdB_1751-2366_ was used as an antigen (Ag) for llama immunization with the schedule shown. FCA, Freund’s complete adjuvant; FIA, Freund’s incomplete adjuvant. (C) ELISA showing the binding response from pre-immune and immune llama sera collected on days 35 and 42 post immunization to coated TcdB_1751-2366_. (D) Serum from day 42 post immunization was fractionated using protein G and protein A affinity columns and analyzed by SDS-PAGE under reducing (R) conditions. G1, A1 and A2 fractions contain heavy-chain IgG (hcIgG, *) while the G2 fraction contains conventional IgG (cIgG, **). The arrows denote IgM heavy and light chains in the A2 fraction. (E) ELISA showing the binding response of fractionated day 42 serum to coated TcdB_1751-2366_. (F) Vero cell cytotoxicity assay demonstrating the effect of fractionated day 42 serum on TcdB inhibition, 72 h post addition of TcdB and polyclonal antibodies to Vero cell monolayers. TcdB was used at 10 and 30 pM and polyclonal fractionated sera at 1 μM. (G, *top*) Summary of solution-phase library panning titers using off-rate selection. In each round, the amount of target (biotinylated TcdB_1751-2366_) was reduced, the amount of competitor (“Comp”; unlabeled TcdB_1751-2366_) was held constant, and the incubation time of phage + target + competitor was increased. After incubation, the complex of phage and biotinylated TcdB_1751-2366_ was captured on streptavidin coated microtiter plates, washed and eluted. (G, *bottom*) Summary of solid-phase library panning titers using coated TcdB_1751-2366_. (H) Phage ELISA showing binding of phage displayed V_H_Hs to coated TcdB_1751-2366_. V_H_Hs on phage producing the highest ELISA signals were expressed, purified and characterized. The Western blot (WB) shows detection of purified V_H_Hs with an α-His6-AP secondary antibody.

In an attempt to generate TcdB neutralizing V_H_Hs, we started by immunizing a llama with recombinant TcdB_1751-2366_ which consists of a portion of the central delivery/translocation domain and entire CROPs domain (**Fig 1A and 1B**). Serum drawn 35 days and 42 days post immunization showed a clear response to TcdB_1751-2366_ by ELISA (**Fig 1C**). Serum drawn on day 42 was separated by fractionation into conventional IgG (cIgG; G2 fraction) and heavy-chain IgG (hcIgG; G1 fraction–a long hinge isotype based on SDS-PAGE, and A1 and A2 fractions–a short hinge isotype(s) based on SDS-PAGE, **Fig 1D**) and showed a typical reactivity pattern by TcdB_1751-2366_ binding in ELISA (**Fig 1E**). The G2 fraction (cIgG) showed the strongest reactivity towards TcdB_1751-2366_ with an EC_50_ ~ 0.1 μg/mL compared to the G1 fraction (hcIgG) with an EC_50_ ~ 2 μg/mL. A1 and A2 fractions (hcIgG) possessed considerably lower TcdB binding titers. We next examined if the fractionated polyclonal sera could neutralize TcdB in Vero cell cytotoxicity assays. TcdB (10 or 30 pM, final) was added to Vero cells alone or in combination with fractionated serum (1 μM, final) for 72 h before addition of WST-1 reagent (**Fig 1F**). At 30 pM of TcdB the G2 fraction fully inhibited TcdB, while approximately 30% was inhibited by the A2 fraction, 10% by the G1 fraction and no inhibition with A1. A similar pattern was seen when 3-fold less TcdB was used: near complete inhibition with G2 and A2, approximately 30% by G1 and less than 10% by A1. The inhibition pattern largely matches the TcdB binding data (**Fig 1E**), although the A2 heavy-chain fraction showed greater inhibition than G1, presumably due to the presence of minor IgM contaminants (**Fig 1D**) that would efficiently neutralize TcdB due to size and valency. We next proceeded with phage display library construction and created a phage display library with a size of ~ 3 x 10^7^ independent transformants.

### V_H_H isolation and expression

For the isolation of V_H_Hs, two different selection strategies were used: solution panning (off-rate based selection) and solid-phase panning (**Fig 1G**). In off-rate based selections 5 nM of biotinylated TcdB_1751-2366_ (**Fig 1A**) was incubated with the phage-displayed V_H_H library followed by addition of a 500-fold molar excess of non-biotinylated TcdB_1751-2366_ (2.5 μM) for 10 min before capture on streptavidin coated wells, washing and elution in round 1 (**Fig 1G**). In each subsequent round the amount of biotinylated TcdB_1751-2366_ was decreased and the incubation time with non-biotinylated TcdB_1751-2366_ competitor increased. For solid-phase selection, TcdB_1751-2366_ was coated directly on standard microtiter plate wells, incubated with library phage and eluted with high pH. In each round of panning the amount of TcdB_1751-2366_ coated was reduced. Monoclonal phage ELISA was performed on clones derived from both methods after three or four rounds of panning and those producing the highest ELISA signals were sequenced and sub-cloned for expression in *E*. *coli* (**Fig 1H**). A total of 29 unique V_H_H sequences (all differing in CDR3; **S1 Table**) were expressed and purified by immobilized metal-ion affinity chromatography (IMAC) with purification yields ranging from 3.1–41.3 mg/L before extensive biophysical characterization.

### Biophysical characterization of V_H_Hs

**Fig 2:**
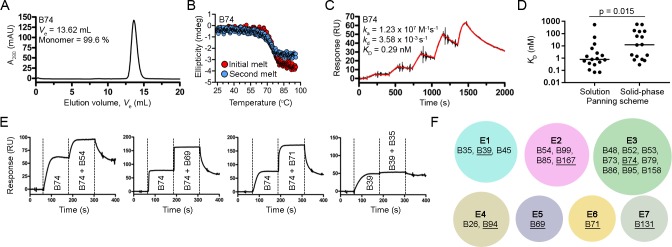
Biophysical characterization of V_H_Hs. Representative SEC profile (A) and thermal unfolding curve from initial and refolded thermal melts (B). (C) Representative SPR single-cycle kinetics sensorgram showed high-affinity binding of V_H_Hs to biotinylated TcdB_1751-2366_ immobilized on a CAP sensor chip. (D) Plot comparing *K*_D_s of V_H_Hs isolated from solution and solid-phase panning schemes. The four V_H_Hs isolated from both panning schemes were included in the analyses. Bars represent the median *K*_D_ and a *P*-value < 0.05 was considered significant (Mann Whitney two-tailed unpaired *t*-test). (E) Representative sensorgrams demonstrating SPR-based epitope binning. All V_H_Hs were injected at 10x *K*_D_ concentrations. (F) Summary of the TcdB_1751-2366_ epitope bins identified in this study by the pool of V_H_Hs tested. The V_H_Hs in each epitope (E) bin are noted and the underlined V_H_Hs represent the highest affinity and/or slowest dissociating antibodies in each bin.

Size-exclusion chromatography (SEC) profiles of the V_H_Hs showed predominantly single monodispersed peaks devoid of higher order aggregates as expected (**Table 1, Fig 2A and S1 Fig**). Circular dichroism spectroscopy was used to determine V_H_H melting temperatures (*T*_m_s) and to assess V_H_H refolding (**Table 1, Fig 2B and S2 Fig**). The *T*_m_s ranged from 57.6 to 87.2°C (median *T*_m_ = 73.4°C) and most V_H_Hs could refold, although the completeness of refolding was sequence dependent. V_H_H binding affinities and kinetics were determined by SPR single-cycle kinetic analysis (**Table 1, Fig 2C and S3 Fig**). V_H_H affinities (*K*_D_s) ranged from 70 pM to 576 nM and, when classified by selection method (**Fig 2D**), the 18 V_H_Hs isolated from solution panning were of statistically higher affinity than the 15 V_H_Hs obtained by solid-phase panning (median *K*_D_s of 0.79 nM and 12.3 nM, respectively, and note that the four V_H_Hs found by both selection methods were counted in each). Next, V_H_Hs with *K*_D_s of ~50 nM and stronger were subjected to epitope binning by SPR-based co-injection (V_H_H 1 followed by V_H_H 1 + V_H_H 2) experiments (**Table 1, Fig 2E and S4 Fig**). From the 21 V_H_Hs binned a total of seven non-overlapping TcdB epitopes were found (**Fig 2F**). The nine V_H_Hs residing in epitope bin E3 were clonally related (**Fig 2F, S1 Table**) while the other six bins contained predominantly unique, unrelated V_H_Hs. Finally, using V_H_Hs with the highest affinities and/or slowest off-rates (*k*_d_s) in each epitope bin (B39, B69, B71, B74, B94, B131 and B167), we examined the TcdB neutralization capacity in Vero cell assays. At the highest V_H_H concentration tested (1 μM) none of the antibodies were capable of inhibiting the cytotoxic effects of TcdB on cells at 1 pM.

**Table 1:** Biophysical properties of anti-TcdB V_H_Hs.

V_H_H	Panning source	*V*_e_ (mL)	SEC (%)^a^	*T*_m_ (°C)^b^	*k*_a_ (M^-1^ s^-1^)	*k*_d_ (s^-1^)	*K*_D_ (nM)	*R*_max_ (RU)^c^	Epitope bin
B26	solution	12.97	99.8	68.3±0.3	5.25×10^6^	3.45×10^−2^	6.3	149	4
B35	solution	14.07	99.8	76.2±0.4	1.16×10^6^	1.05×10^−3^	0.9	50	1
B39	solution	16.05	99.8	87.2±0.2	3.44×10^7^	2.56×10^−3^	0.07	43	1
B45	solution	14.84	99.4	79.5±0.4	3.52×10^6^	3.84×10^−4^	0.1	55	1
B46	solid phase	13.18	99.3	61.0±1.4	1.53×10^5^	9.43×10^−3^	62	51	n.d.
B48	both	13.07	99.7	82.5±0.3	4.11×10^6^	5.71×10^−3^	1.4	73	3
B52	both	12.77	99.9	65.3±0.7	1.29×10^7^	1.01×10^−2^	0.8	63	3
B53	solid phase	12.97	99.6	77.1±0.0	4.38×10^6^	7.98×10^−3^	1.8	72	3
B54	solution	12.85	95.3	81.7±0.3	3.86×10^7^	1.47×10^−2^	0.4	65	2
B55	solid phase	13.35	99.8	66.6±1.1	3.34×10^5^	4.93×10^−2^	147	142	n.d.
B56	solid phase	14.00	86.7	57.6±0.3	5.46×10^4^	3.15×10^−2^	576	18	n.d.
B65	solid phase	13.26	96.3	84.4±0.2	1.32×10^4^	2.33×10^−3^	176	17	n.d.
B69	solid phase	13.11	82.1	80.9±0.5	1.17×10^7^	1.50×10^−1^	12.8	102	5
B71	solid phase	12.88	85.9	63.3±0.3	7.76×10^6^	9.18×10^−2^	11.8	61	6
B73	both	13.41	99.7	80.2±0.0	1.27×10^7^	9.19×10^−3^	0.7	63	3
B74	both	13.62	99.6	75.0±0.2	1.23×10^7^	3.58×10^−3^	0.3	66	3
B76	solid phase	12.48	99.4	84.2±1.1	1.38×10^6^	2.17×10^−1^	157	74	n.d.
B79	solid phase	13.48	99.1	66.9±0.1	5.59×10^6^	9.72×10^−3^	1.7	75	3
B85	solution	12.94	99.4	69.7±0.1	1.93×10^7^	1.14×10^−2^	0.6	73	2
B86	solution	13.03	99.6	72.9±0.1	8.25×10^6^	3.98×10^−3^	0.5	71	3
B92	solid phase	12.85	98.9	78.0±0.1	1.51×10^6^	8.28×10^−1^	548	72	n.d.
B94	solution	12.58	96.7	69.7±0.4	6.19×10^5^	8.77×10^−3^	14.2	385	4
B95	solid phase	13.97	95.1	80.8±0.6	1.12×10^7^	7.05×10^−3^	0.6	72	3
B96	solution	13.17	99.7	70.5±0.2	1.37×10^6^	7.33×10^−2^	53.6	104	n.d.
B99	solution	13.05	98.7	69.8±2.2	6.33×10^7^	1.04×10^−1^	1.6	76	2
B131	solution	13.01	100	69.3±2.3	5.47×10^7^	1.40×10^−1^	2.6	73	7
B149	solution	13.45	97.4	83.9±0.0	4.12×10^4^	2.20×10^−2^	534	133	n.d.
B158	solution	13.44	99.4	73.4±0.8	3.44×10^4^	1.74×10^−3^	50.5	67	3
B167	solution	13.14	99.6	72.1±0.4	1.53×10^7^	1.10×10^−3^	0.07	76	2

^**a**^% monomer determined by area under the curve

^b^(mean ± SD)

^c^Observed *R*_max_ was obtained from a TcdB surface with a theoretical *R*_max_ of 200 RUs; n.d., not determined.

### Reformatting V_H_Hs as dimeric molecules

**Fig 3:**
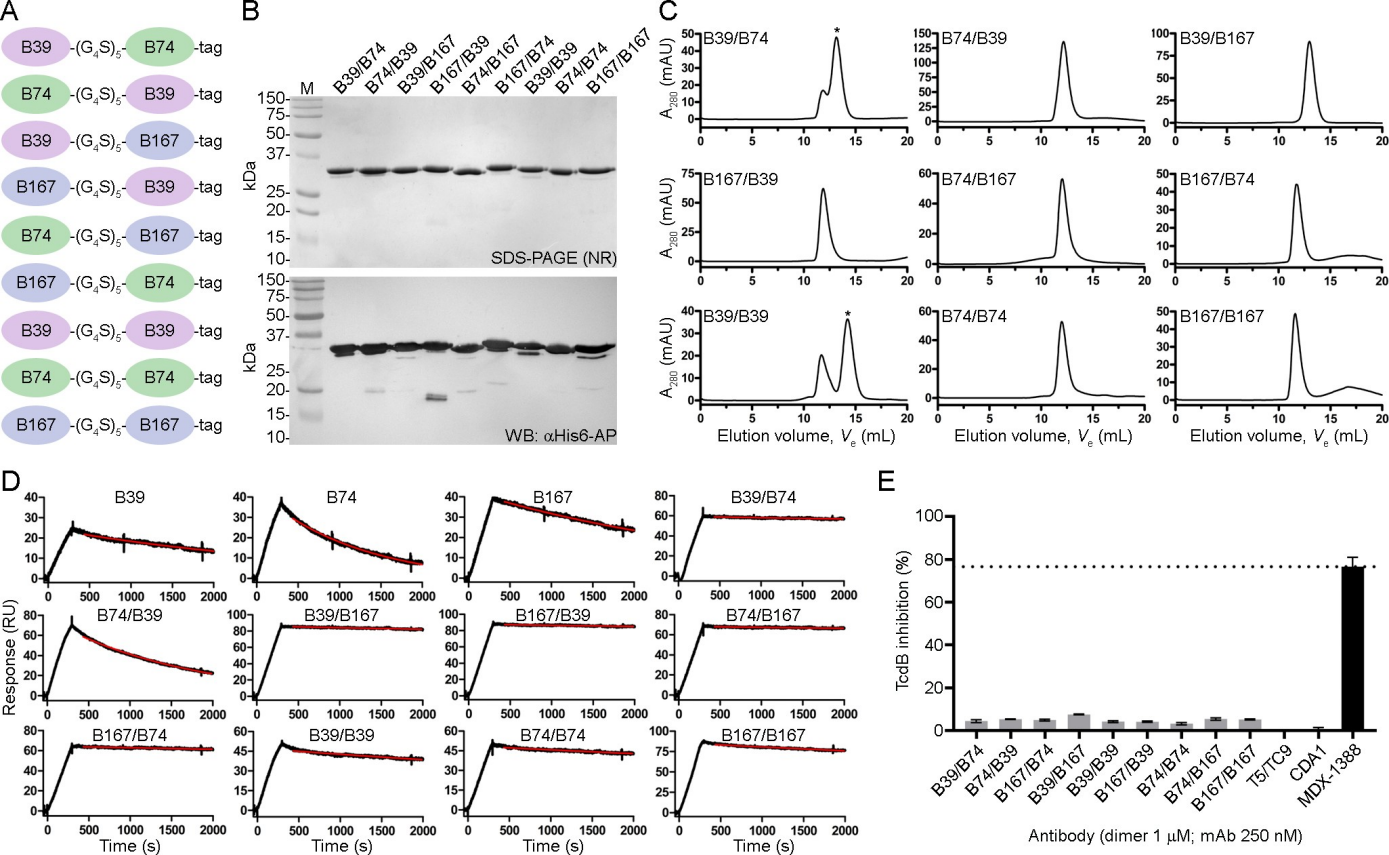
Generation and characterization of V_H_H-V_H_H dimers. (A) Cartoon diagram of V_H_H-V_H_H dimers separated by a 25 amino acid linker (G_4_S)_5_ and containing a C-terminal His_6_ “tag”. (B) Dimers were expressed, purified and analyzed by non-reducing (NR) SDS-PAGE and probed by α-His_6_-AP in Western blot (WB). M, protein molecular mass marker. (C) SEC profiles of dimers. Asterisks denote the peaks that were selected for SPR analysis when aggregates were present. (D) SPR sensorgrams showing dissociation phases of V_H_H-V_H_Hs and parent monomers. (E) TcdB neutralization with V_H_H-V_H_H dimers. The final TcdB concentration of 3–10 pM and V_H_H-V_H_H concentration of 1 μM was co-incubated with Vero cell monolayers for 72 h before addition of WST-1 cytotoxicity reagent. MDX-1388 was used at 250 nM. Neutralization values are presented as mean ± SD from n = 3 independent experiments.

We next generated V_H_H-V_H_H dimers using the three highest affinity V_H_Hs that targeted unique epitopes (B39, B74 and B167) to determine if biparatopic designs could impart a measureable level of TcdB neutralization not seen with V_H_H monomers. All possible combinations of the three antibodies were created including homodimers (**Fig 3A**), with a standard 25 amino acid linker separating each V_H_H. Dimers were expressed in *E*. *coli*, purified by IMAC with yields ranging from 2.0 to 12.0 mg/L (**Fig 3B**) and assessed by SEC (**Fig 3C**) which revealed a predominantly single monodispersed species with the exception of B39/B74 and B39/B39 dimers that showed higher order aggregates. V_H_H-V_H_H dimer *T*_m_s ranged from 64.4 to 73.1°C. SPR off-rate analysis demonstrated nearly irreversible bivalent binding to TcdB_1751-2366_ surfaces for many of the dimers, approaching the instrument limit of detection (**Table 2, Fig 3D**). Vero cell neutralization assays using the dimers showed minor TcdB inhibition with all nine formats tested ranging from 3.2% (B39/B39) to 7.5% (B167/B39) maximum inhibition (**Table 2, Fig 3E**). The negative control T5/TC9 dimer did not inhibit TcdB and the benchmark control mAb MDX-1388 reached a maximum inhibition of 76.6%.

**Table 2:** Biophysical properties of anti-TcdB V_H_H-V_H_H dimers.

V_H_H- V_H_H	Yield (mg/ L)^a^	*V*_e_ (mL)^b^	*T*_m_ (°C)^c^	*k*_d_ (s^-1^)	Maximum TcdB neutralization (%)^c^^,^^d^
B39/B74	9.2	13.2	71.6 ± 0.2	2.2 × 10^−5^	4.4 ± 0.4
B74/B39	3.6	12.2	70.9 ± 1.6	5.9 × 10^−4^	5.3 ± 0.1
B39/B167	9.0	13.0	73.1 ± 1.7	2.4 × 10^−5^	4.9 ± 0.3
B167/B39	2.0	11.9	67.7 ± 1.4	1.7 × 10^−5^	7.5 ± 0.1
B74/B167	5.8	12.0	64.4 ± 2.1	1.4 × 10^−5^	4.2 ± 0.2
B167/B74	2.4	11.7	66.5 ± 0.6	2.4 × 10^−5^	4.1 ± 0.2
B39/B39	12.0	14.2	79.5 ± 3.4	9.7 × 10^−5^	3.2 ± 0.3
B74/B74	2.2	12.0	70.4 ± 3.3	6.3 × 10^−5^	5.4 ± 0.3
B167/B167	6.8	11.6	67.5 ± 2.3	6.0 × 10^−5^	5.1 ± 0.2
MDX-1388	-	n.d.	n.d.	n.d.	76.6 ± 4.4^e^

^a^Purification yield from 1 L *E*. *coli* culture

^b^*V*_e_ from Superdex 200

^c^(mean ± SD)

^d^1 μM or

^e^250 nM antibody + 3 pM TcdB vero cell cytotoxicity (72 h); n.d., not determined.

### Reformatting V_H_Hs as Fc fusions

**Fig 4:**
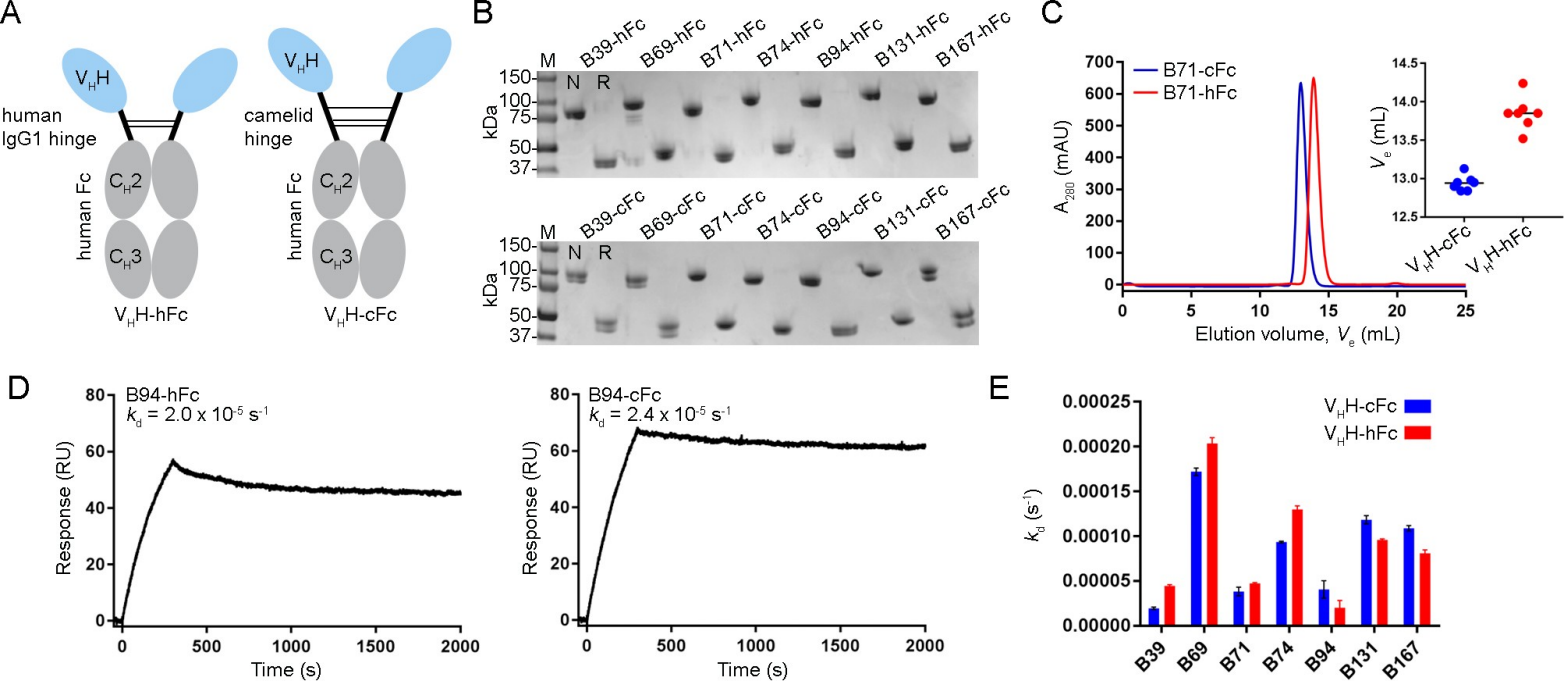
Generation and characterization of V_H_H-Fcs with human or camelid hinges. (A) Cartoon diagram illustrating the two V_H_H-Fc formats used with varying hinge length and composition. (B) Purified V_H_H-Fcs were analyzed by SDS-PAGE under non-reducing (N) and reducing (R) conditions. (C) Representative V_H_H-Fc SEC elution profile from a Superdex 200 column. *Inset*, plot of SEC elution volumes for all V_H_H-cFcs and V_H_H-hFcs. (D) Representative SPR sensorgrams showing 30 min (1800 s) dissociations of V_H_H-Fc fusions flowing over immobilized TcdB_1751-2366_ surfaces. (E) Dissociation rate comparison of V_H_H-Fcs with human or camelid hinges.

Given the modest level of TcdB inhibition seen with the V_H_H-V_H_H dimers, we next explored if construction of larger molecules may lead to greater TcdB neutralizing potency. V_H_H-Fc fusions (**Fig 4A**) were constructed using each of the seven high-affinity V_H_Hs from distinct epitope bins (B39, B69, B71, B74, B94, B131 and B167). The designs consisted of a set of V_H_H-Fcs with a 15-residue human IgG1 hinge (EPKSCDKTHTCPPCP) and another set of complementary molecules with a 35 residue camel/llama γ2a hinge (EPKIPQPQPKPQPQPQPQPKPQPKPEPECTCPKCP), to explore the possible advantages a longer, more flexible hinge may have on TcdB inhibition. The V_H_H-Fcs, denoted “V_H_H-hFc” for molecules containing the human hinge and “V_H_H-cFc” for molecules containing the camel hinge, were expressed in mammalian cells and purified by protein A with yields ranging from 2.5 to 21.6 mg/100 mL of culture (**Table 3, Fig 4B**). SEC analyses of V_H_H-Fcs revealed single, monodispersed peaks and consistently showed V_H_Hs with camel hinges eluting earlier, most likely due to their larger hydrodynamic radius (**Table 3, Fig 4C and S5A Fig**). SPR-determined off-rates demonstrated nearly irreversible bivalent binding to TcdB_1751-2366_ surfaces (**Table 3, Fig 4D and S5B Fig**). Comparison of the off-rates (*k*_d_s) of V_H_H-Fcs with human or camel hinges did not reveal significant differences in their ability to bind TcdB_1751-2366_ (**Fig 4E**).

**Table 3:** Biophysical properties of anti-TcdB V_H_H-Fc fusions.

V_H_H-Fc/mAb	Hinge	Yield (mg/ 100 mL)^a^	*V*_e_ (mL)^b^	*k*_d_ (s^-1^)	Maximum TcdB neutralization (%)^c^^,^^d^
B39-hFc	human	5.7	17.64	4.5 × 10^−5^	0.0
B69-hFc	human	13	14.24	2.0 × 10^−4^	18.0 ± 10.2
B71-hFc	human	6	13.91	4.7 × 10^−5^	11.0 ± 7.0
B74-hFc	human	4.4	13.82	1.3 × 10^−4^	26.3 ± 4.5
B94-hFc	human	12.2	13.52	2.0 × 10^−5^	55.9 ± 22.4
B131-hFc	human	14.6	13.70	9.6 × 10^−5^	18.0 ± 13.4
B167-hFc	human	2.5	13.85	8.1 × 10^−5^	37.5 ± 2.7
B39-cFc	camelid	7.9	15.07	2.0 × 10^−5^	0.1 ± 0.1
B69-cFc	camelid	9.1	13.13	2.0 × 10^−4^	20.2 ± 5.7
B71-cFc	camelid	11.3	12.98	4.7 × 10^−5^	8.9 ± 5.5
B74-cFc	camelid	6.8	12.95	9.4 × 10^−5^	22.3 ± 4.2
B94-cFc	camelid	11.8	12.84	2.4 × 10^−5^	61.2 ± 1.3
B131-cFc	camelid	21.6	12.84	9.6 × 10^−5^	18.7 ± 12.3
B167-cFc	camelid	9.2	12.90	8.1 × 10^−5^	22.8 ± 2.9
MDX-1388	human	-	n.d.	n.d.	70.7 ± 13.9

^a^Purification yield from 100 mL HEK293 culture

^b^*V*_e_ from Superdex 200

^c^(mean ± SD)

^d^250 nM antibody + 500 fM TcdB vero cell cytotoxicity (72 h); n.d., not determined.

### TcdB neutralization with V_H_H-Fcs

**Fig 5:**
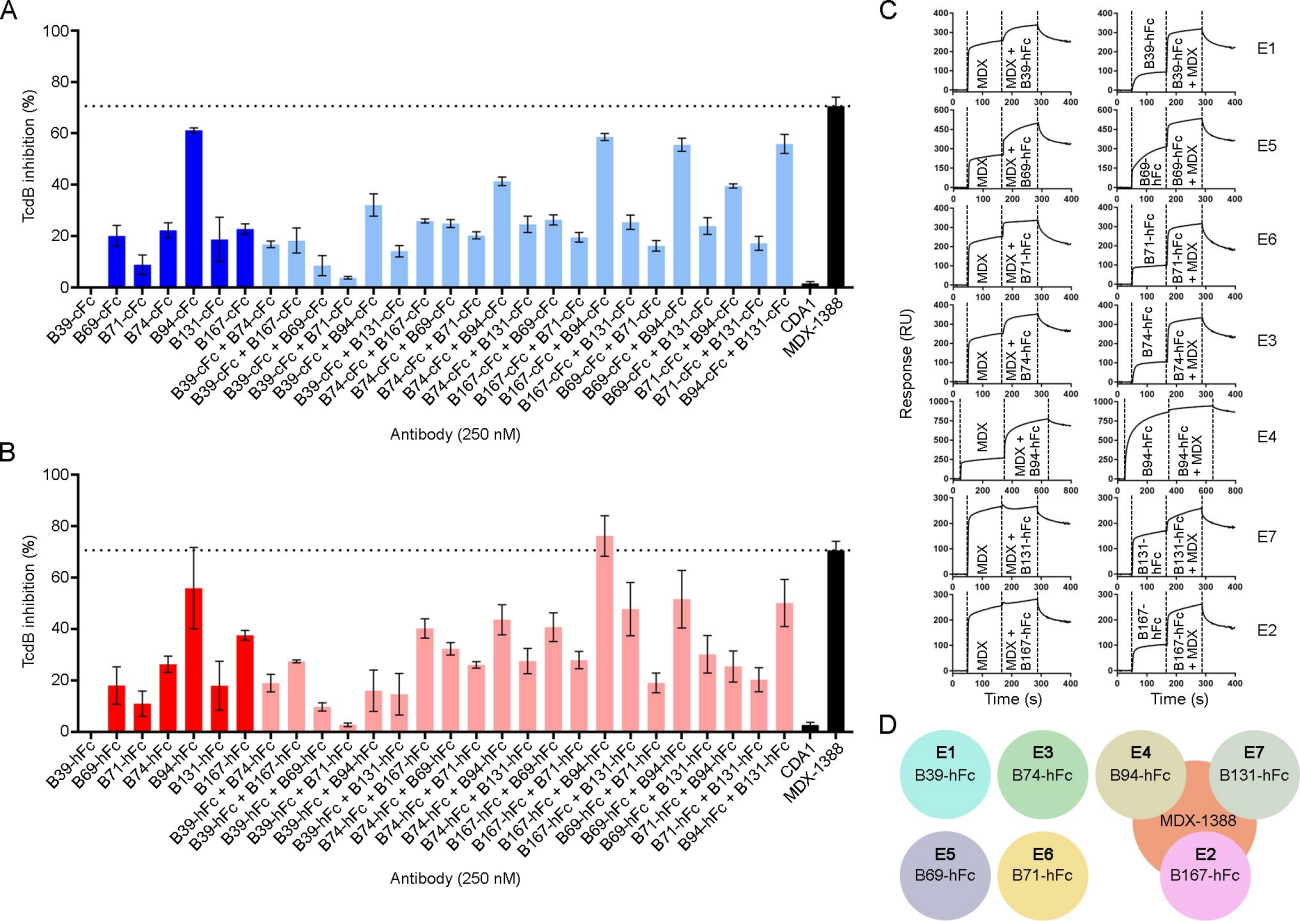
TcdB neutralization assays with V_H_H-Fcs. (A) TcdB neutralization with V_H_H-Fcs containing a camelid hinge. (B) TcdB neutralization with V_H_H-Fcs containing a human hinge. In (A) and (B), the final TcdB concentration of 500 fM and final antibody concentration of 250 nM (single antibody) or 125 + 125 nM (pairs) were co-incubated with Vero cell monolayers for 72 h before addition of WST-1 cytotoxicity reagent. Neutralization values are presented as mean ± SD from n = 4 independent experiments. (C) Sensorgrams showing SPR-based epitope binning of MDX-1388 mAb with each V_H_H-hFc, in both orientations, with antibodies injected at 25–50 × *K*_D_ concentrations over TcdB_1751-2366_ surfaces. Epitope bins corresponding to each V_H_H-hFc are noted. (D) Summary of V_H_H-Fc reactivity to TcdB_1751-2366_ illustrating that several V_H_H-Fcs bind TcdB at sites distinct from MDX-1388, while others bind TcdB at regions that partially overlap with MDX-1388.

The neutralization potencies of V_H_H-Fcs were compared to the benchmark mAb bezlotoxumab (MDX-1388) in TcdB inhibition assays using 500 fM TcdB and 250 nM antibody (**Table 3, Fig 5A and 5B**). MDX-1388 showed a maximum TcdB inhibition of 70.7% compared to V_H_H-Fcs that ranged from 8.9% (B71-cFc) to 61.2% (B94-cFc). There were essentially no differences between the neutralizing capacities of V_H_H-Fcs containing the short IgG1 hinge or the longer camel hinge, mirroring the near identical off-rates determined by SPR. Neither format of B39-Fc neutralized TcdB. CDA1 (an anti-TcdA mAb) [36] was included as a negative control and did not neutralize TcdB as expected. To examine possible synergistic effects from combinations of V_H_H-Fcs on TcdB inhibition, pairs of V_H_H-Fcs (125 nM + 125 nM) were examined in neutralization assays (**S2 Table, Fig 5A and 5B**). The V_H_H-Fc pair of B94-hFc + B167-hFc achieved a maximum TcdB inhibition of 76.2%, slightly exceeding the neutralization potency of each V_H_H-Fc alone and that of MDX-1388. In pairs containing B39-Fc, the non-neutralizing antibody, overall neutralization was reduced by approximately 50% of the maximum inhibiting potency of the second antibody partner, reflecting the fact that 50% less inhibitory antibody was present (**Fig 5A and 5B**).

### V_H_H-Fc competition with bezlotoxumab

To determine if our panel of neutralizing V_H_H-Fcs recognized similar or unique TcdB epitopes from that of bezlotoxumab we performed SPR co-injection experiments (**Fig 5C and 5D**). B39-hFc, B69-hFc, B71-hFc and B74-hFc did not compete with MDX-1388 in either injection sequence, indicating that the four V_H_H-Fcs bind sites on TcdB completely independent of the MDX-1388 binding site. This is unsurprising for B39-hFc given the inability of this antibody to neutralize TcdB. The other three V_H_Hs were previously shown to bind unique epitopes as monomers (**Fig 2F**) suggesting B69-hFc, B71-hFc and B74-hFc recognize three novel TcdB epitopes that support neutralizing antibodies. The results of B94-hFc, B131-hFc and B167-hFc binning with MDX-1388 revealed significant overlap in TcdB binding patterns. When MDX-1388 was injected first, B94-hFc was partially blocked by pre-bound MDX-1388 and B131-hFc and B167-hFc were completely blocked by pre-bound MDX-1388. In the opposite orientation, MDX-1388 binding was completed blocked by pre-bound B94-hFc, and partially blocked by pre-bound B131-hFc or B167-hFc. Collectively this data suggests the most potent TcdB neutralizing V_H_H-Fcs (B94-hFc, B167-cFc) bind TcdB at sites that partially overlap with the MDX-1388 binding site at the N-terminal end of the CROPs domain [27].

## Discussion

In this work we set out to identify high-affinity V_H_Hs capable of neutralizing *C*. *difficile* TcdB. We previously failed to identify monomeric V_H_H neutralizers when immunizing with a small C-terminal fragment of the CROPs domain [10]. Here our expanded immunogen design containing a portion of the central delivery domain and the entire CROPs domain yielded a number of V_H_Hs that were capable of TcdB inhibition when formatted as dimers and more so as Fc fusions. It should be noted that our previous TcdB-binding V_H_Hs [10] were not tested as V_H_H-Fc fusions and may have been capable of TcdB inhibition, although their affinities were considerably weaker than the V_H_Hs isolated in this work. Once again the monomeric V_H_Hs did not inhibit TcdB, suggesting a steric element that is required for TcdB inhibition when targeting the CROPs domain. Consistent with our findings, Yang *et al* isolated several TcdB-binding V_H_Hs and found five high-affinity V_H_Hs targeting the C-terminal CROPs domain that failed to inhibit TcdB cytotoxicity while those targeting the N-terminal GTD domain were potent neutralizers [13].

Our work is not the first to report TcdB-inhibiting V_H_Hs binding the CROPs domain. Andersen *et al* isolated several inhibitory monomeric V_H_Hs binding this domain, indicating that TcdB neutralization is possible with monomeric antibodies [15]. It is not clear how they successfully identified V_H_Hs that recognize critical epitope(s) for TcdB function/cell binding, that were not identified here, in our early work [10] or by [13], while employing a similar immunization strategy. Interestingly two of their non-neutralizing CROPs-binding V_H_Hs were converted into inhibitory antibodies when displayed on the surfaces of lactobacilli [15], again pointing to a large steric element in imparting TcdB inhibition with antibodies binding this region of the toxin.

Beyond V_H_Hs there are several potent TcdB-neutralizing mAbs that have been well studied. The most advanced anti-TcdB mAb is bezlotoxumab which received FDA approval for recurrent *C*. *difficile* infection in 2016. This antibody was originally isolated in a study reported by [36] and demonstrated to bind the C-terminal receptor binding domain (CROPs domain). Structural studies performed by [27] revealed the precise binding site of the mAb lies within the first two of four CROP repeat domains, with each CROP domain consisting of three short repeating units (SRs) followed by one long repeating unit (LR) and two more SRs. SPR binding data showed bezlotoxumab bound two distinct epitopes which was later supported by X-ray crystal structures and homology modeling showing two Fab fragments binding adjacent to each other in the N-terminal half of the CROPs domain (aa 1834–2101). Given bezlotoxumab neutralizes TcdB by preventing binding to mammalian cells [27,37], one can assume that some of our most potent V_H_H-Fc fusions (B94-Fc, B131-Fc and B167-Fc) neutralize TcdB in a similar manner given that they partially overlap with bezlotoxumab for TcdB binding. Whether the exact mechanism of inhibition is due to blocking putative carbohydrate binding site interactions with a host-cell receptor, or by steric effects precluding the central/delivery domain from making contacts with FZD2, PVRL3 or CSPG4 receptors is unknown. It is interesting to note that the observed *R*_max_ of B94 in SPR experiments was considerably higher than other antibodies as both a V_H_H monomer (**Table 1**) and as a V_H_H-Fc in binning experiments (**Fig 5C**), suggesting that B94 may bind to repeating TcdB CROPs domain epitopes. Supporting this idea is the fact that B94-Fc was the only V_H_H-Fc to completely block MDX-1388 binding when B94-Fc was bound to TcdB first. If a repeating epitope is bound by B94-Fc, it would suggest a similar mechanism of inhibition to that of MDX-1388 and that affinity improvements may lead to greater neutralizing potency since the monovalent affinity of B94 is relatively weak (*K*_D_ = 14 nM) compared to a Fab fragment from MDX-1388 (*K*_D_ = 19 pM or 370 pM; depending on the TcdB epitope) [27].

For the other three neutralizing V_H_H-Fcs described here (B69-Fc, B71-Fc and B74-Fc) their location for toxin binding and subsequently their mechanism for toxin inhibition also remain unknown. B39, which failed to neutralize TcdB as an Fc fusion and did not overlap with bezlotoxumab as expected, was previously [12] co-crystalized with a C-terminal fragment of the TcdB CROPs domain (aa 2248–2367 of TcdB from strain 10463) and definitively showed only recognition of a single, non-repeating TcdB epitope. This may suggest that antibodies binding at a distance from the central delivery/translocation domain have minimal effects on TcdB inhibition compared to antibodies binding nearby in the N-terminal half of the CROPs domain. Elsewhere, [28] have successfully identified four inhibitory mAbs targeted to the CROPs domain of TcdB that were neutralizers alone and to a greater extent when combined in pairs. Additionally, TcdB-neutralizing mAbs recognizing the C-terminal GT domain of TcdB have proven to be both potent inhibitors and effective in *in vivo* protection assays [38,39,40].

There were several other interesting observations of note from this work. The conventional IgG (cIgG) fraction obtained from llama serum post immunization with TcdB_1751-2366_ was capable of inhibiting TcdB in cytotoxicity assays more efficiently than the three heavy-chain IgG (hcIgG) fractions. While the cIgG binding titer for TcdB was higher than the best hcIgG fraction by approximately 10-fold, which is a typical binding pattern we have observed in several other immunization campaigns, there were dramatic differences in the inhibition pattern seen between G1 (hcIgG) and G2 (cIgG) fractions. It is possible that a greater agglutination mechanism with camelid cIgGs compared to camelid hcIgGs is at play here and could be due to the physical distance separating the binding arms of each antibody format. We hypothesize that the more compact hcIgG footprint may bias the polyclonal pool toward intra-toxin binding events and that the greater distance afforded to cIgGs promotes both intra-toxin and inter-toxin binding events, leading to increased agglutination and ultimately greater TcdB inhibition. Supporting this is the fact the A2 (hcIgG) fraction, which showed a much weaker TcdB binding titer than the G1 fraction, possessed considerably greater neutralizing potency likely driven by IgM antibodies found as contaminants in the fractionated serum.

We performed panning experiments in solution using off-rate based selection and this method produced statistically higher affinity V_H_Hs compared to panning on immobilized antigen. This method of selection was also the source of the V_H_H-Fcs with the highest neutralizing potency, even though two of the three most potent TcdB inhibitors did not possess the highest overall monovalent V_H_H affinities. It is probable that the solution panning scheme presented the randomly biotinylated TcdB in a more native-like conformation and made more areas of the protein available for V_H_H binding compared to selection of V_H_Hs on TcdB-coated wells. This may explain the higher average binding affinity and greater neutralization potency of V_H_Hs isolated by the solution panning approach. We also examined the use of a longer camel hinge in place of the human IgG1 hinge to present the V_H_H in a more natural context when tethered to the Fc domain. While SEC profiles clearly showed that V_H_H-Fcs with camel hinges eluted earlier and thereby suggest a larger hydrodynamic volume, similar dissociation rate constants and TcdB neutralizing potency demonstrated there was no clear benefit to using the longer hinge. We do not completely understand why this was the case but speculate that the camel hinge only moderately expands the binding distance between V_H_H arms, still less than the footprint of a cIgG, and that neutralization is dependent on combination of factors including the location, geometry and accessibility of TcdB epitopes.

In summary, the V_H_Hs described here were potent TcdB neutralizing antibodies on par with bezlotoxumab when formatted as Fc fusions. We envision creating even more effective TcdB-neutralizing agents through optimization of affinities and binding geometries, such as through structure-guided biparatopic designs. Combining anti-TcdB biparatopic V_H_H-V_H_H designs with TcdA-neutralizing V_H_Hs onto a human Fc scaffold would allow for the generation of ultra-potent toxin inhibitors in a single antibody format, similar to approaches described previously [13,17], while maintaining the steric requirements for TcdB neutralization and long serum half-life. The *in vivo* efficacy previously demonstrated with multivalent designs that include linking anti-TcdA and anti-TcdB V_H_Hs suggests the inclusion of GTD-targeting anti-TcdB antibodies is critical [13,17,18]. Our results make a case for targeting the central delivery and CROPs domains with V_H_Hs. Whether including CROPs-targeting V_H_Hs in these designs will be as potent as those targeting the GTD region remains to be seen. In addition, finer epitope mapping of the V_H_H-Fcs that bound TcdB at regions distinct from bezlotoxumab will reveal if these antibodies recognize the central delivery domain or the CROPs domain and the nature of these unique inhibitory epitopes. Finally, the V_H_Hs described here will serve as useful additions to the reagent toolkit for further refinement of the mechanism of TcdB-host cell interactions.

## Supporting information

S1 Fig SEC chromatograms of V_H_H monomers. V_H_Hs were passed over a Superdex 75 column at a flow rate of 0.5 mL/min in HBS-EP buffer. Molecular mass standards are shown. The percent monomer was calculated from the peak area under the curve. *V*_e_, elution volume.
(TIF)

S2 Fig Thermal unfolding of V_H_H monomers by circular dichroism spectroscopy. (A) Thermal unfolding of V_H_Hs (50 μg/mL, 3.2 μM) was performed in phosphate buffer and measured in a 5 mm cuvette at 215 nm. V_H_Hs were allowed to cool at 25°C for 3 h before the second thermal unfolding (refolded melting curve) was performed. (B) Voltage comparing two V_H_Hs (B54 and B99) that aggregate as a consequence of unfolding versus one V_H_H that does not (B53).
(TIF)

S3 Fig Single-cycle kinetics sensorgrams showing monomeric V_H_Hs binding to TcdB_1751-2366_ surfaces. Black lines represent raw data and red lines represent 1:1 binding model fits. Rate constants and affinities are shown for each antibody. The irrelevant V_H_H served as a negative control. For experimental conditions see Materials and methods.
(TIF)

S4 Fig Representative sensorgrams from SPR-based epitope binning of V_H_H monomers on TcdB_1751-2366_ surfaces. The first V_H_H was injected at 10× *K*_D_ concentration followed immediately by injection of a mixture of the first V_H_H + second V_H_H at 10× *K*_D_ concentration. For experimental conditions see Materials and methods.
(TIF)

S5 Fig Characterization of V_H_H-Fc fusions. (A) SEC chromatograms of V_H_H-Fcs on a Superdex 200 column at a flow rate of 0.5 mL/min in HBS-EP buffer. (B) SPR sensorgrams illustrating the dissociation of 1 nM V_H_H-Fcs from TcdB_1751-2366_ surfaces. For experimental conditions see Materials and methods.
(TIF)

S1 Table Clonal relatedness of TcdB-specific V_H_Hs isolated in this study. (PDF)

S2 Table Select pairs of VHH-Fcs with the highest TcdB neutralization. (PDF)
