# Supplementary figures and images for: Neutralization of Clostridium difficile toxin B with VHH-Fc fusions targeting the delivery and CROPs domains
Source: PLoS One. 2018 Dec 12;13(12):e0208978. doi: 10.1371/journal.pone.0208978 (PMC6291252; doi:10.1371/journal.pone.0208978)

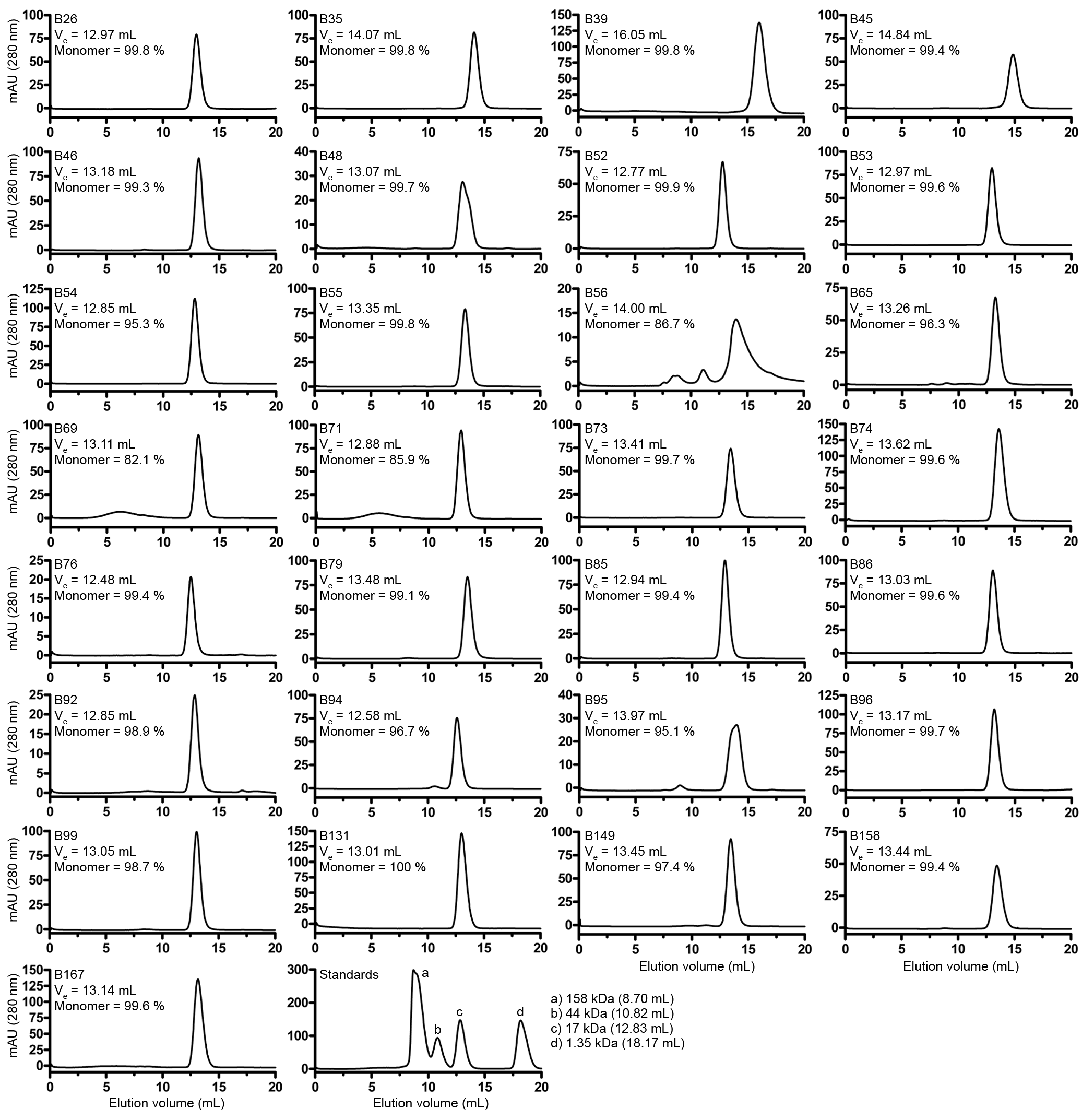

Supplement: S1 Fig — VHHs were passed over a Superdex 75 column at a flow rate of 0.5 mL/min in HBS-EP buffer. Molecular mass standards are shown. The percent monomer was calculated from the peak area under the curve. Ve, elution volume. (TIF) [file pone.0208978.s001.tif]

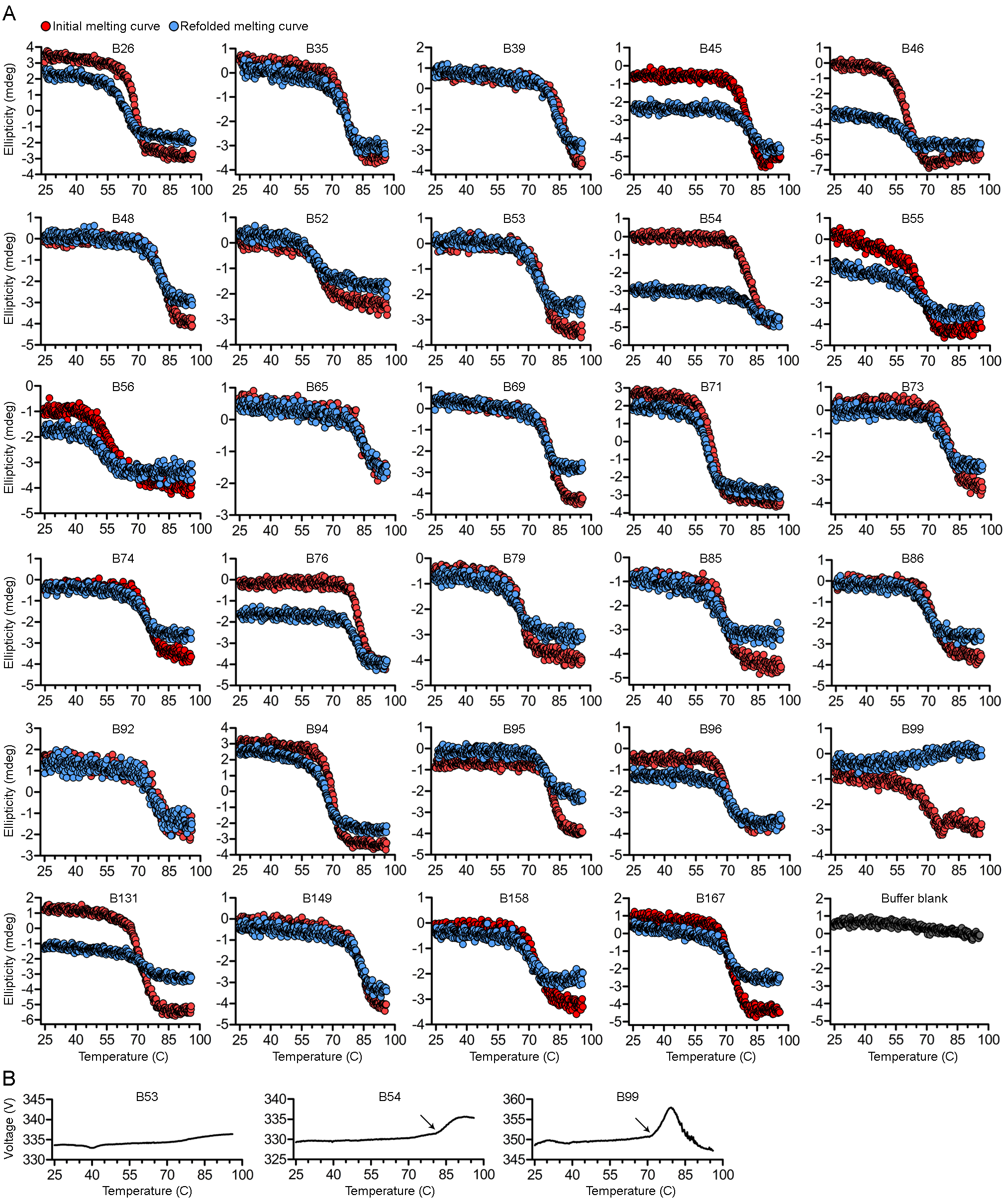

Supplement: S2 Fig — (A) Thermal unfolding of VHHs (50 μg/mL, 3.2 μM) was performed in phosphate buffer and measured in a 5 mm cuvette at 215 nm. VHHs were allowed to cool at 25°C for 3 h before the second thermal unfolding (refolded melting curve) was performed. (B) Voltage comparing two VHHs (B54 and B99) that aggregate as a consequence of unfolding versus one VHH that does not (B53). (TIF) [file pone.0208978.s002.tif]

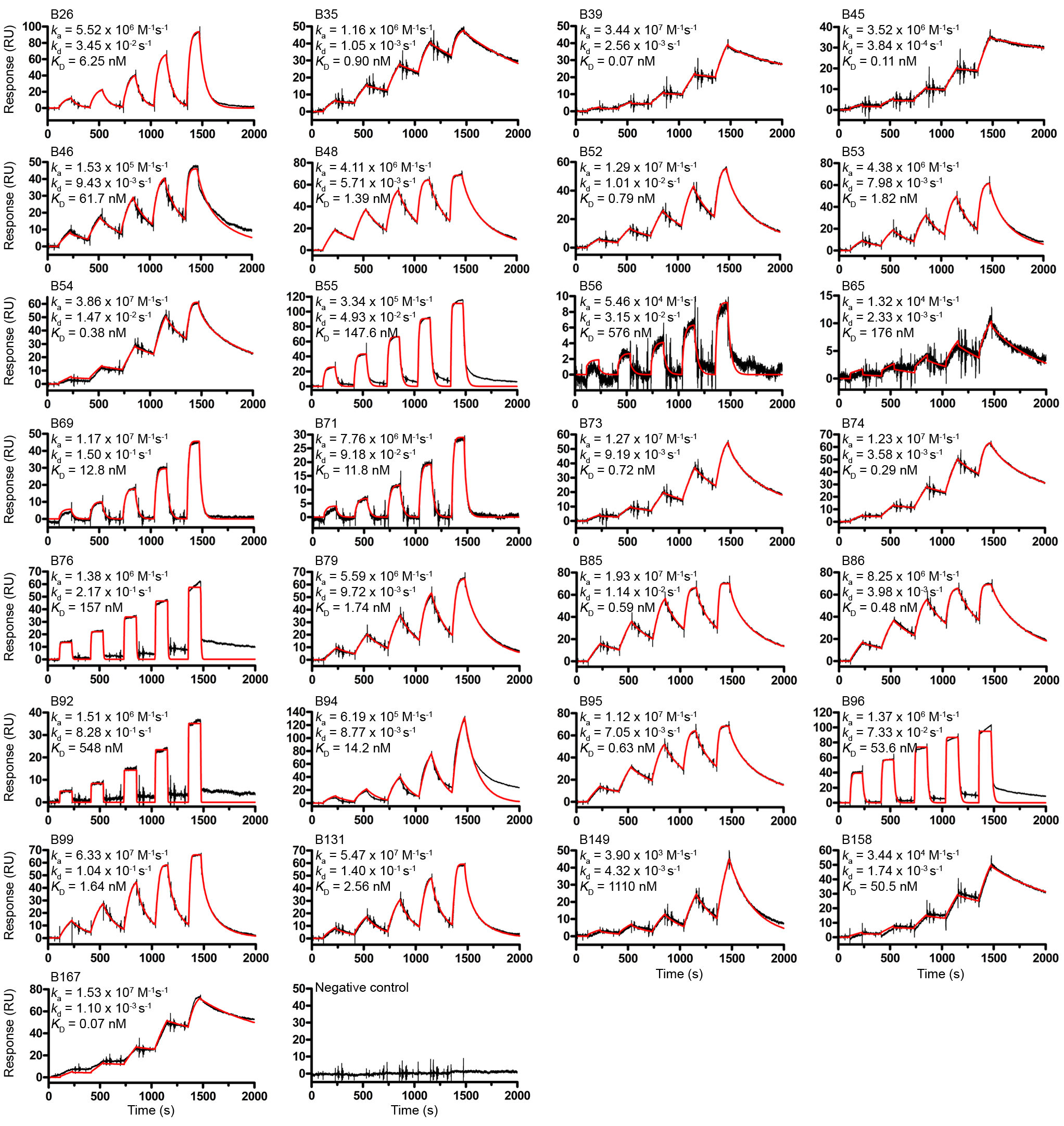

Supplement: S3 Fig — Black lines represent raw data and red lines represent 1:1 binding model fits. Rate constants and affinities are shown for each antibody. The irrelevant VHH served as a negative control. For experimental conditions see Materials and methods. (TIF) [file pone.0208978.s003.tif]

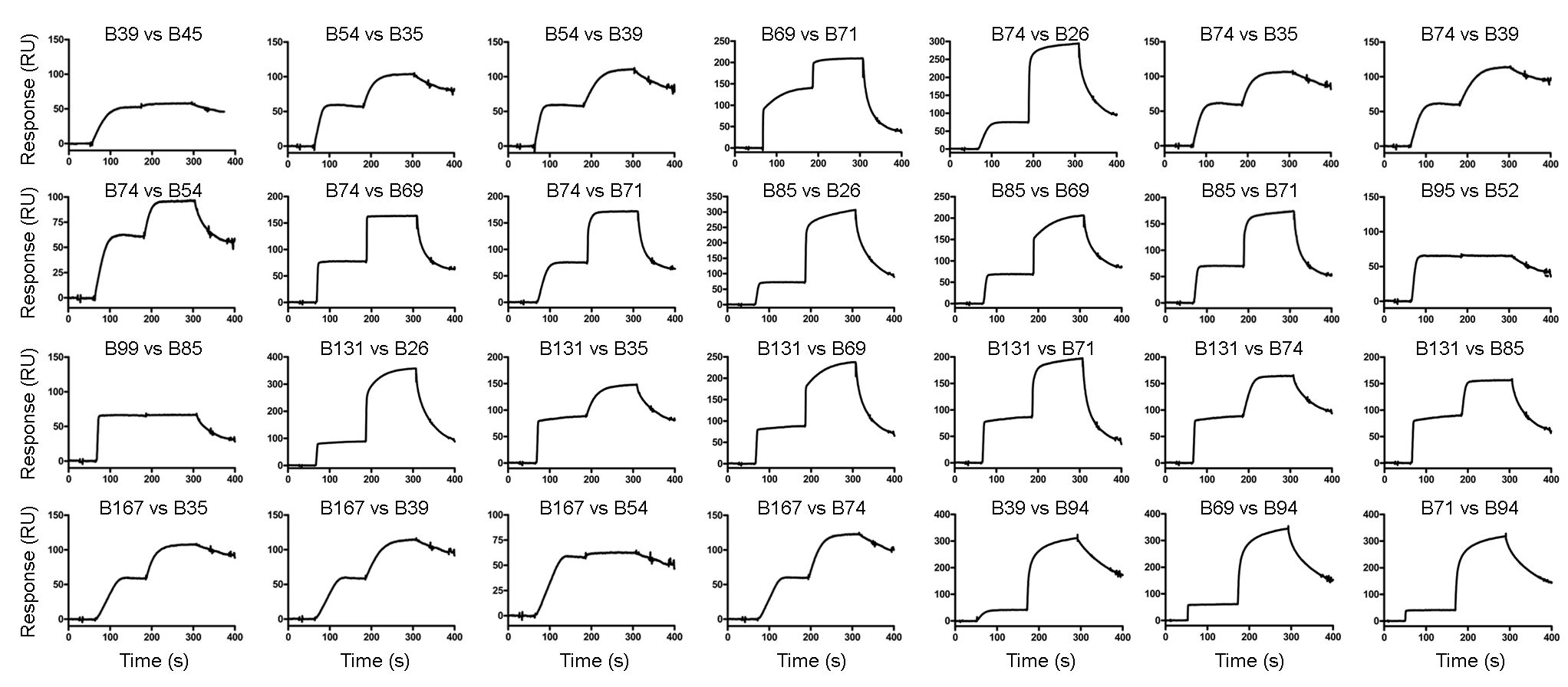

Supplement: S4 Fig — The first VHH was injected at 10× KD concentration followed immediately by injection of a mixture of the first VHH + second VHH at 10× KD concentration. For experimental conditions see Materials and methods. (TIF) [file pone.0208978.s004.tif]

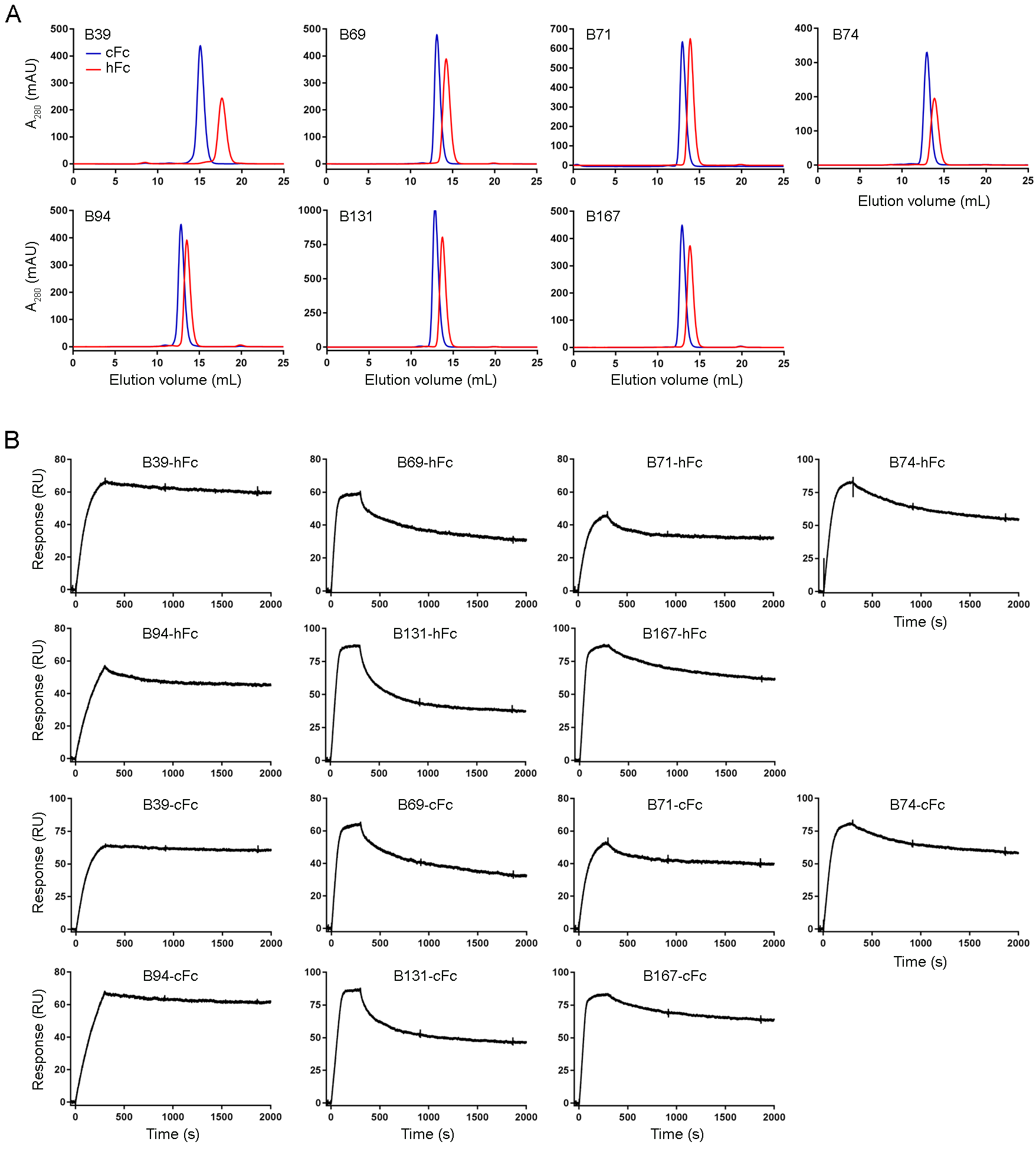

Supplement: S5 Fig — (A) SEC chromatograms of VHH-Fcs on a Superdex 200 column at a flow rate of 0.5 mL/min in HBS-EP buffer. (B) SPR sensorgrams illustrating the dissociation of 1 nM VHH-Fcs from TcdB1751-2366 surfaces. For experimental conditions see Materials and methods. (TIF) [file pone.0208978.s005.tif]
