# Supplementary material for: Neutralization of Clostridium difficile toxin B with VHH-Fc fusions targeting the delivery and CROPs domains
Source: PLoS One. 2018 Dec 12;13(12):e0208978. doi: 10.1371/journal.pone.0208978 (PMC6291252; doi:10.1371/journal.pone.0208978)
Supplement: S1 Table — (PDF) [file pone.0208978.s006.pdf]

S1 Table: Clonal relatedness of TcdB-specific V_HHs isolated in this study.

Epitope bin	Clone group	V_HH	CDR3 length	Comment
1	a	B35	17	
	b	B39	16	B39 and B45 differ at 1 position in CDR2 and 1 position in CDR3
		B45	16	
2	c	B54	19	B54 and B99 differ at 1 position in CDR3
		B99	19	
	d	B85	19	B85 and B167 differ at 1 position in CDR3
		B167	19	
3	e	B48	20	
		B52	20	[#]Differs at 1 position in CDR1 and 2 positions in CDR3
		B53	20	[#]Differs at 1 position in CDR1, 1 position in CDR2 and 3 positions in CDR3
		B73	20	[#]Differs at 1 position in CDR1 and 2 positions in CDR3
		B74	20	[#]Differs at 2 positions in CDR1 and 2 positions in CDR3
		B79	20	[#]Differs at 2 positions in CDR1 and 3 positions in CDR3
		B86	20	[#]Differs at 1 position in CDR1 and 3 positions in CDR3
		B95	20	[#]Differs at 1 position in CDR1 and 3 positions in CDR3
		B158	20	[#]Differs at 2 positions in CDR1 and 3 positions in CDR3
4	f	B26	22	B26 and B94 differ at 2 positions in CDR1 and 3 positions in CDR3
		B94	22	
5	g	B69	18	
6	h	B71	20	
7	i	B131	16	
n.d.	j	B92	22	B92 and B149 differ at 3 positions in CDR1, 5 positions in CDR2 and 1 position in CDR3
		B149	22	
n.d.	k	B46	17	
n.d.	l	B55	16	
n.d.	m	B56	8	
n.d.	n	B65	18	
n.d.	o	B76	19	
n.d.	p	B96	22	

CDRs are defined according to IMGT (www.imgt.org)

[#]relative to B48 V_HH
