# Supplementary material for: Neutralization of Clostridium difficile toxin B with VHH-Fc fusions targeting the delivery and CROPs domains
Source: PLoS One. 2018 Dec 12;13(12):e0208978. doi: 10.1371/journal.pone.0208978 (PMC6291252; doi:10.1371/journal.pone.0208978)
Supplement: S2 Table — (PDF) [file pone.0208978.s007.pdf]

Table S2: Select pairs of V_HH-Fcs with the highest TcdB neutralization.

**V_HH-Fc 1**	**V_HH-Fc 2**	**Epitope bins**	**Maximum TcdB neutralization (%)[a,b]**
B69-hFc	B74-hFc	5 + 3	32.3 ± 4.8
B69-hFc	B94-hFc	5 + 4	51.6 ± 22.4
B69-hFc	B167-hFc	5 + 2	40.7 ± 11.2
B74-hFc	B94-hFc	3 + 4	43.6 ± 11.6
B74-hFc	B167-hFc	3 + 2	40.2 ± 7.5
B94-hFc	B131-hFc	4 + 7	50.2 ± 18.3
B94-hFc	B167-hFc	4 + 2	76.2 ± 15.8
B131-hFc	B167-hFc	7 + 2	47.7 ± 20.8
B69-cFc	B94-cFc	5 + 4	55.6 ± 5.1
B71-cFc	B94-cFc	6 + 4	39.5 ± 1.7
B74-cFc	B94-cFc	3 + 4	41.4 ± 3.3
B94-cFc	B131-cFc	4 + 7	55.9 ± 7.5
B94-cFc	B167-cFc	4 + 2	58.6 ± 2.7
MDX-1388[c]			70.7 ± 13.9

[a](mean ± SD)

[b]125 nM V_HH-Fc 1 + 125 nM V_HH-Fc 2 + 500 fM TcdB vero cell cytotoxicity (72 h)

[c]250 nM of mAb.
